# Mouse Models of Antigen Presentation in Hematopoietic Stem Cell Transplantation

**DOI:** 10.3389/fimmu.2021.715893

**Published:** 2021-09-14

**Authors:** Motoko Koyama, Geoffrey R. Hill

**Affiliations:** ^1^Clinical Research Division, Fred Hutchinson Cancer Research Center, Seattle, WA, United States; ^2^Division of Medical Oncology, University of Washington, Seattle, WA, United States

**Keywords:** transplantation, antigen presentation, allogeneic stem cell transplantation, graft-*versus*-host disease, graft-*versus*-leukemia effects

## Abstract

Allogeneic stem cell transplantation (alloSCT) is a curative therapy for hematopoietic malignancies. The therapeutic effect relies on donor T cells and NK cells to recognize and eliminate malignant cells, known as the graft-versus-leukemia (GVL) effect. However, off target immune pathology, known as graft-versus-host disease (GVHD) remains a major complication of alloSCT that limits the broad application of this therapy. The presentation of recipient-origin alloantigen to donor T cells is the primary process initiating GVHD and GVL. Therefore, the understanding of spatial and temporal characteristics of alloantigen presentation is pivotal to attempts to separate beneficial GVL effects from detrimental GVHD. In this review, we discuss mouse models and the tools therein, that permit the quantification of alloantigen presentation after alloSCT.

## Introduction

Allogeneic stem cell transplantation (alloSCT) remains a curative therapy for a broad range of hematopoietic malignancies including acute myeloid leukemia (AML) and myelodysplastic syndrome. The therapeutic effect largely resides in graft-versus-leukemia (GVL) effects where graft-derived donor T cells and NK cells recognize allogeneic, hematopoietic or tumor-associated antigens. Unfortunately, this process is closely related to adverse immune effects, namely graft-versus-host disease (GVHD), where donor T cells attack normal recipient tissue. To date, the separation of beneficial GVL from detrimental GVHD remains the greatest unmet need in alloSCT.

The immunological pathways of both GVL and GVHD are initiated by the presentation of allogeneic antigens to donor T cells: Autologous or syngeneic (from an identical twin donor) transplants do not induce classical GVL or GVHD due to the lack of alloreactivity (1). AlloSCT using rigorous T cell-depletion (TCD) prevents severe GVHD but increases leukemia relapse (2–4), indicating that the recognition of alloantigens by the donor T cell is essential in disease pathophysiology. Therefore, studies to elucidate potential spatial and temporal differences in antigen presentation within GVL and GVHD have been undertaken (i.e. what types of antigen presenting cells (APC) and donor T cell subsets are involved, in which organs, and at what time after transplant?).

When choosing mouse models to study GVHD, at least three factors should be considered. Firstly, who are the cellular mediators of disease? As we previously reviewed (5, 6), both donor CD4 and CD8 T cells which recognize alloantigens presented by MHC class II (MHC-II) and class I (MHC-I) respectively, can mediate distinct patterns of GVL and GVHD. Host APC initiate both GVHD and GVL, in contrast, donor APC predominantly invoke GVHD but not GVL (7–9). To define the specific pathways of MHC-I *vs* MHC-II dependent T cell GVHD and the donor *vs*. host APC involved, alloantigen-specific T cell receptor (TCR) transgenic T cells are useful tools, particularly when combined with mutant mice bearing defined genetic defects in antigen presentation. Secondly, donor T cell responses can generally be initiated by one of two types of alloantigen presentation. One is the presentation of host minor histocompatibility antigens (miHAs) derived from polymorphic proteins which are present in recipients but not donors (10, 11). A miHA is presented by MHC which is shared by the donor and the host, and donor T cells recognize miHAs in the same way as pathogen-derived antigens are seen. This process includes a process known as epitope or antigen spreading whereby T cells recognize a family of antigens that have diversified from the original parental epitope (12). This explains why HLA-matched unrelated donors are at a higher risk for GVHD than HLA-matched sibling donors (13), since the frequency of genetic disparity for individual miHA is generally two-fold higher (10, 14). The other type of antigen recognition by the donor T cell is within the complex of mismatched MHC and a non-polymorphic peptide. Naive T cells have been educated in the thymus to react a peptide loaded on self MHC. Therefore, donor T cells are not designed to react to antigen presented by a mismatched MHC. However, a scenario can occur whereby the molecular complex of a peptide and mismatched MHC is structurally sufficiently similar to that of another peptide and matched MHC to activate a donor T cell. This type of donor T cell antigen recognition happens in MHC-mismatched transplantation and is known as molecular mimicry (15). This process explains why increased numbers of mismatched MHC loci (6/8, 7/8 *vs* 8/8 HLA match) significantly increases GVHD and decreases overall survival regardless of the underlying type of malignant disease (16). Thirdly, the pathophysiology and manifestations of GVHD seen after transplant should recapitulate those in clinical GVHD. The pathways of antigen presentation leading to GVHD are highly promiscuous in xenograft systems where immune deficient mice (and their APC) stimulate a human T cell response, such that their usefulness in studying the mechanisms of GVHD is somewhat limited.

Many non-transgenic donor and host combinations have been well established for the study of GVHD. These include MHC mismatched or MHC-matched but miHA-mismatched models which are typically dominated by MHC-I or II dependent GVHD that is largely strain dependent. We direct the reader to excellent reviews on the subject of these non-transgenic models (17). Here we focus on antigen-specific models of GVHD.

## Alloantigen Presentation in Models of GVHD Targeting Minor Histocompatibility Antigens

In MHC matched systems, the cognate recognition of antigen by a mature, thymically educated, donor T cell requires the TCR to interact with host polymorphic peptide (miHA) presented by a HLA molecule common to donor and host. Defined human miHAs have been the subject of recent reviews (10, 11, 14). While the most common molecular mechanism generating miHAs is single nucleotide polymorphisms (SNPs) within gene exons that modify peptide binding to MHC or TCR, other mechanisms such as altered protein transport/processing or transcription can also cause the generation of dramatically new epitopes (10, 11). The expression of ovalbumin (Ova) in BMT recipients under control of ubiquitous (e.g. β-actin) promoters may mimic the latter setting. Since ova is not expressed by normal mice, transgenic ova production by recipient mice can be a dominant antigen to CD4^+^ and CD8^+^ T cells (18, 19). When ova expression is limited to specific cell types such as hematopoietic cells (20, 21) or leukemic cells (22), it may mimic hematopoiesis-specific or leukemia-specific miHA. Given that these hematopoietic- or leukemia-restricted miHAs have attracted attention as targets for clinical TCR transgenic T cell therapy (23, 24), these antigen model systems can be useful in understanding immunity within these contexts. In regard to ova, extensive tool reagents are available. Ova peptide-specific CD4^+^ and CD8^+^ TCR transgenic mice (OT-II, DO11.10 and OT-I mice) can be utilized as a source of donor T cells, whereby short term T cell activation and expansion can be used to quantify antigen presentation (18, 19, 25). Ova peptide-MHC tetramers can be utilized to detect peptide-specific T cells within polyclonal T cells (26) and the monoclonal antibody (25-D1.16) can also quantify ova-peptide loaded within MHC -I to quantify direct antigen presentation (27). Many foreign peptide/proteins other than ova, such as virus-derived proteins, can also be exploited in a similar fashion (**Table 1**).

**Table 1:** Minor antigens within MHC matched systems and antigen-specific TCR transgenic T cells (top), peptide-MHC tetramer to detect antigen-specific T cells (middle) and antibodies to quantify antigen-MHC complexes (bottom).

Ag protein	Ag peptide	MHC-restriction		TCR-Transgenic mouse		Reference	
Ova albumin	OVA257-264 (SIINFEKL)	H-2K^b^		OT-I		(19)	
H60	LTFNYRNL	H-2K^b^		J15		(28, 29)	
H-Y, *Uty* gene	WMHHNMDLI	H-2D^b^		MataHari		(30)	
H-Y	unknown	H-2D^b^		HY-TCR		(31)	
H-Y	unknown	I-A^b^		Rachel		(32)	
H-Y, *Dby* gene	NAGFNSNRANSSRSS	I-A^b^		Marilyn		(33, 34)	
I-E	Eα52-68	I-A^b^		TEa		(35)	
Ova albumin	OVA323-339	I-A^b^		OT-II		(18)	
Ova albumin	OVA323-339, 327-333, 328-338	I-A^d^		DO11.10		(25)	
Ag protein	Ag peptide	MHC-restriction		TCR Detection tetramer		Reference	
H60	LTFNYRNL	H-2K^b^		H6/H-2K^b^ tetramer		(36, 37)	
H-Y, *Uty* gene	WMHHNMDLI	H-2D^b^		HY-Uty/H-2D^b^ tetramer		(37)	
Ova albumin	OVA323-339	I-A^b^		OVA323-339/I-A^b^ tetramer		(38)	
Ova albumin	OVA323-339	I-A^d^		OVA323-339/I-A^d^ tetramer		(26)	
Ag protein	Ag peptide	MHC-restriction		Antibody reactivity		Clone	Reference
Ova albumin	OVA257-264 (SIINFEKL)	H-2K^b^		Against SIINFEKL bound to H-2K^b^		25-D1.16	(27)
I-Ea chain	Eα52-68	I-A^b^		Against Eα52-68 peptide bound to I-A^b^		Y-Ae	(39, 40)
hen egg lysozyme (HEL)	HEL-derived peptide (HEL48-62)	I-A^k^		Against HEL peptide (residue 48-62) bound to I-A^k^		AW3.18	(41)

Superscript letters indicate MHC types.

Similarly, strain-specific models of endogenous antigen also exist. H60 protein, a ligand for NKG2D, is expressed by hematopoietic cells but not parenchyma cells in a strain-specific manner. H60 is expressed by BALB.B mice but not C57Bl6 (B6) nor C3H.SW mice, and these three stains are all MHC-matched (H-2^b^) but miHA disparate. Recently, H60 transduction has been undertaken into B6-background leukemia or recipient mice and H60 peptide-H2K^b^ tetramers used to detect responding CD8^+^ T cells in combination with MHC-I deficient mice (H-2K^b-/-^) to demonstrate how defects in leukemia antigen presentation promote exhaustion of donor T cells and ineffective GVL (42) (**Table 1**).

HLA molecules are highly polymorphic (43). Since HLA-mismatched transplants (including haplo-identical transplantation) are all semi HLA-matched, mismatched HLA-derived peptides can be presented by another shared HLA. To mimic this scenario, TEa transgenic TCR (Vα2/Vβ6) T cells and YAe antibody recognize the same complex of mismatched MHC-derived peptide presented within MHC-II (Eα52-68 peptide and I-A^b^ respectively) (35, 39, 40). B6 mice lack I-Eα chain, hence, do not express I-E, a MHC-II locus, whereas many other strains express the I-Eα chain. Thus the Eα52-68 peptide derived from the I-Eα chain of relevant recipient strains can stimulate TEa T cells and bind to the YAe antibody in an I-A^b^-restricted manner. To our knowledge, YAe, the aforementioned 25-D1.16 and AW3.18 which binds to hen egg lysozyme (HEL) peptide loaded on I-A^k^, are the only antibodies that bind to specific peptide-MHC complexes that are commercially available (**Table 1**). They are highly useful tools for the quantification of antigen presentation. Thus while antigen-specific T cell expansion detected by tetramer or as TCR transgenic T cell expansion reflects overall antigen presentation, these antibodies allow direct quantification of antigen presentation within individual APC subsets that are distinguishable by flow cytometry (e.g. donor cell *vs*. host cells, dendritic cell subsets *vs* macrophages, and within different organs) (44).

The transplantation of a female-derived graft into a male recipient is a known risk factor for GVHD (13). Multiple H-Y antigens encoded by Y-chromosome genes have been identified (e.g. SMCY, UTY, DBY, DEFRY) and reactive T cell clones have been isolated from female transplant recipients rejecting male grafts and male recipients transplanted with female grafts (45–48). Multiple murine TCR clones and TCR transgenic lines reactive to H-Y antigens (e.g. UTY, DBY) have been generated on a B6 background (**Table 1**) (30–34). In addition to their clear clinical relevance, these systems allow the use of male B6 mice from most transgenic and mutant strains (e.g. MHC-deficient recipients) to delineate mechanistic pathways of antigen presentation. As such, these systems provide powerful tools for the study of GVHD. The incorporation of reporter constructs such as luciferase into these TCR transgenic systems allows detailed and tissue specific compartmentalization of antigen presentation (20, 44, 49, 50).

## Alloantigen Presentation in Models Characterized by Molecular Mimicry

It is well established in studies some 50 years ago that 1 - 10% peripheral T cells are reactive to non-self (mismatched) MHC, although the frequency of T cells that can respond to self (matched) MHC-expressing cell loaded with foreign Ag is likely at least 100-fold lower (51, 52). However, the mechanism underlying the high degree of clonal T cell alloreactivity to MHC-mismatched antigen has only recently been elucidated (53, 54). For decades there was a controversy over whether a T cell reacts to peptide-alloMHC (mismatched MHC) complexes in a peptide-centric or MHC-centric manner. In the former, TCR primarily interacts with the peptide rather than mismatched MHC, whereas the latter anticipates that a TCR primarily recognizes structural determinants on the (mismatched) MHC structure (15, 54, 55). The dispute has now been settled in favor of reactivity against the hybrid of peptide- and MHC-centric hypothesis. A TCR can thus recognize peptide-loaded allogeneic MHC 1) in docking modes disparate to those that are germline-encoded following thymic education (55) and 2) in the germline-encoded mode *via* molecular mimicry whereby the TCR binds to very similar structure formed by a foreign peptide presented on self-MHC and an endogenous peptide presented on allogeneic MHC (15). Both theories potentially explain allogeneic MHC reactivity. The former scenario of disparate docking modes has been demonstrated for 2C TCR (H-2K^b^) T cells which react to a self-peptide (dEV8, also known as Ndufa4_54–61_) derived from enzyme NADH-ubiquinone oxidoreductase, loaded on H-2K^b^ (56, 57) and indeed this clone has been used to study positive selection in the thymus (57, 58). This scenario is of questionable relevance to transplant immunology where non-self-reactive mature T cells recognize MHC-mismatched cells. In the latter setting, what is striking is the demonstration that a TCR clone (LC13) recognizing Epstein-Barr virus (EBV)-derived peptide on self-MHC (HLA-B*0801) can recognize self-peptide on some allogeneic MHCs (HLA-B*4402 and B*4405, but not B*4403) due to a similar conformation (molecular mimicry) after TCR ligation (15). HLA-B*4402 or B*4405 transfected HLA class I-deficient (C1R) cell lines (C1R-B*4402 and C1R-B*4405) but not a B*0801 transfected one (C1R-B*0801) can activate LC13, indicating that endogenous antigens (e.g. ATP-binding cassette protein) can stimulate TCR clones when they are presented by some but not all allogeneic HLA molecules. Vice versa, an EBV-peptide can stimulate LC13 when it is presented by self-HLA (B*0801) but not allogeneic HLA (B*4405). Indeed, healthy individuals that are heterozygous for HLA-B*0801 and B*4402 do not possess this dominant LC13 TCR clonotype, demonstrating this clonotype has been clonally deleted due to potential self-reactiveness, and instead, they generate alternative clonotypes reactive to the same viral epitope (59). This suggests a phenomenon whereby one TCR clone reactive to a foreign peptide also responds to endogenous peptides presented by other MHC molecules within one individual. In the MHC-mismatched allogeneic transplant a donor TCR repertoire will encounter new MHC molecules, and be activated by host mismatched MHC molecules loaded with endogenous (non-polymorphic) peptides [a schematic illustration depicting the different modes of alloantigen presentation has been published previously (5)].

To study alloantigen presentation by a mismatched MHC molecule, MHC-mismatched models can be chosen [e.g. B6 (H-2^b^) → BALB/c (H-2^d^)]. MHC-partial mismatched or haplo-mismatched models include the possibility that matched MHC molecules present miHAs derived from mismatched MHC molecules. In this context, B6-background Bm1 (MHC-I mutation resulting in amino acid substitution) and Bm12 (MHC-II mutation resulting in amino acid substitution) mice are useful (**Table 2**). When either CD4^+^ or CD8^+^ T cells and BM cells from wild-type B6 were injected into lethally irradiated Bm1 and Bm12 recipients, donor CD4^+^ T cells induced lethal GVHD in only Bm12 recipients, and donor CD8^+^ T cells did so only in Bm1 recipients (67). When Bm12 T cells were transplanted in MHC-II deficient or wild-type B6 mice, serum IFN-γ was elevated in wild-type recipients but not in MHC-II deficient recipients (68). Similarly, *in-vitro* culture (mixed lymphocyte reaction) demonstrated that B6 CD4^+^ T cells proliferate in response to Bm12 cells but not Bm1 or B6 (self) cells, and B6 CD8^+^ T cells proliferate to Bm1 cells but not Bm12 or B6 cells (67). Despite the potential possibility that Bm1 and Bm12 mutation themselves serve as miHAs on conserved MHC-I or II molecules, this scenario would generate both CD4^+^ and CD8^+^ T cell responses, and so can be discounted. Instead, they suggest that both mutated MHC-I and II, H2-K^bm1^ and H2-Ab1^bm12^, are loaded with endogenous peptides that bind B6 CD8^+^ and CD4^+^ TCR repertoires, respectively. There are other many similar MHC-I-mutated mice, most of which have mutation in the H-2K locus (e.g. bm3 and bm8) (69, 70), while MHC-II-mutated strains are limited to Bm12 (**Table 2**).

**Table 2:** Antigen presentation within mismatched MHC. miHA matched.

Mismatched MHC	MHC mutation	Known peptide/MHC complex	T cell’s self-MHC	Reactive TCR Detection by	T cell from	Reference
H-2K^bm1^	7 nucleotides		H-2K^b^		Multiple clones from C57BL6	(60)
H2-Ab1^bm12^	3 nucleotides		H2-Ab1^b^		Multiple clones from C57BL6	(61, 62)
H-2L^d^		dEV8 (self-peptide)/H-2K^b^, dEV8/H-2K^bm3^, SIYR (foreign peptide)/H-2K^b^, p2Ca/H-2L^d^, QL9/H-2L^d^	H-2^b^	1B2 (anti-2C TCR mAb)	2C TCR transgenic mice	Originally BALB.B CD8^+^ T cells when immunized P815 (DBA/2 mastocytoma line) and BALB/c splenocytes (63–65).
H2-IA^d^ (I-A^d^)		unknown non-polymorphic mouse peptide/I-A^d^	H2-Ab1 (I-A^b^)		4C TCR transgenic mice	(66)

Superscript letters indicate MHC types.

In contrast to studies utilizing specific mutations within MHC class I or II, TCR transgenic T cells which react to specific MHC disparities have also been exploited. In addition to the previously described 2C TCR transgenic CD8^+^ T cells, 4C TCR transgenic CD4^+^ T cells from B6 mice respond to an endogenous and ubiquitously expressed mouse non-polymorphic peptide presented on I-A^d^ (66).

## Antigen Presentation in Xenograft Transplant Models

There has been a controversy in regard to how faithfully inbred murine allogeneic transplant models recapitulate GVHD in outbred humans. A number of studies have thus been conducted in xenogeneic transplant systems whereby human hematopoietic cells [most commonly peripheral blood mononuclear cells (PBMC)] are transplanted into severely immunodeficient mice (e.g. NSG, NRG, NOG mice) (71). PBMC is predominantly composed of lymphocytes, although APC including monocytes and dendritic cells are included. However, there is no hematopoietic progenitor or stem cell components, hence, the differentiation of human APC is not sustained. There is also a question as to whether human T cells can appropriately recognize murine MHC and if not whether these systems are indeed clinically relevant. In addition, there are three other major constraints to the interpretation of xenogeneic transplant systems. Firstly, it does not phenocopy clinical GVHD. While clinical acute GVHD typically targets the skin, liver and gastrointestinal (GI) tract and the intestinal disease usually determines lethality, the skin and GI tract display only very mild changes after xenogeneic transplant (72, 73). The major pathogenic manifestations of GVHD in xenogeneic transplant models are predominantly observed in the liver and lung and give rise to lethality. Second, since human cell engraftment is limited and predominantly of T cells after PBMC are transplanted, the GVHD induced is unlikely to recapitulate the spectrum seen following full T and myeloid cell engraftment seen in species-specific systems. Finally, it is unclear the role that mouse anti-human graft rejection (e.g. by myeloid cells) plays in the spectrum of GVHD seen in these systems (72, 74, 75).

It has been demonstrated that murine MHC-I and II molecules stimulate human T cells after human PBMC injection into NSG mice (73, 75, 76). When recipient NSG mice lack murine MHC-I expression, disease lethality and the frequency of human CD3^+^ T cells in the recipient are reduced. The presence or absence of murine MHC-II expression is less important in isolation since its deficiency does not attenuate lethality (75). Although these data suggest that human T cells can react to murine MHC, human T cells primarily respond to human MHC (HLA) molecules rather than murine MHC *in vitro* (77, 78). Therefore, multiple immunodeficient mouse strains expressing HLA class I (e.g. HLA-A2) and class II (e.g. HLA-DR1 or DR4) have been developed (73, 79, 80). These mice have been demonstrated to develop HLA-restricted anti-virus human T cell clones after human HSC transplantation (79, 80), suggesting the transgenic human HLA indeed preferentially invoke human TCR responses. However, physiological upregulation of MHC and antigen presentation therein are not assured. While multiple cytokines (i.e. interferon (IFN)-γ, interleukin (IL)-4, IL-6, IL-10, IFN-α/β and tissue necrosis factor) and glucocorticoids modulate MHC-I and II expression (81, 82) and many are secreted by human T cells after xenogeneic transplantation, the majority are not cross-reactive with the relevant murine receptors.

The presence of both murine and human MHC in these humanized transgenic systems likely creates promiscuous antigen recognition. NSG mice with intact murine MHC (H-2) and transgenic HLA-A*0201 expression develop accelerated lethality after transplantation with human PBMC, but equivalent histopathology (relative to HLA-A*0201-negative NSG mice) (83). *In vitro* assays demonstrate that multiple human CD4^+^ and CD8^+^ T cell clones are reactive to both murine MHC-I and II (84). In addition, highly aberrant CD4^+^CD8^+^ T cell expansion within tissue has been seen after xenogeneic but not clinical transplantation and this likely reflects non-physiological antigen presentation (85). Thus while the mechanism by which human TCR can respond to a murine peptide-MHC complex is an intriguing question, the reality is that disordered antigen presentation is a serious confounding factor.

Another possible approach of *in vivo* model is to utilize the mice which have already been reconstituted by human hematopoiesis. To achieve human hematopoietic APC engraftment, the transplant of human bone marrow or cord blood (CB) derived CD34^+^ HSC into immune deficient mice is promising (74, 86, 87). These methods achieved stable and high level of human cell engraftment in the BM and spleen (> 50%) but with a low frequency of CD33^+^ or CD14^+^ human myeloid cells. Human CB-derived HSC injection into newborn NSG mice demonstrated the presence of human HLA-DR^+^CD11c^+^ cell in the spleen three months after transplant (86). NSG-SGM3 (NSGS) mice which express additional transgenic genes for human IL-3, GM-CSF and SCF and MISTRG mice which express human IL-3, GM-CSF, M-CSF, thrombopoietin and SIRPα, significantly improve human myeloid cell reconstitution (88–90). Nevertheless, the issue of concurrent murine MHC expression in these systems remains a confounding variable.

## Conclusions

The advantage of fully murine models that permit delineation of antigen-specific responses includes the ability to spatially and temporally track antigen presentation and resultant T cell responses *in vivo*, coupled with extensive availability of mutant and transgenic strains to delineate mechanisms of disease. Nevertheless, it remains important to validate these results with polyclonal T cells in MHC-mismatched or miHA-mismatched transplant models. The use of xenograft models are increasingly important for the examination of immune independent therapeutic effects (e.g. the effect of a drug on a human leukemia *in vivo*) or human-human cellular interactions *in vivo* (e.g. a human CAR T cell or TCR transgenic T cell response against a human leukemia). In contrast, the species mismatch inherent in these systems at the APC-T cell interface makes them more problematic as a robust preclinical transplant platform. Hence, the use of xenogeneic models for GVHD/GVL studies ought to be used cautiously, sparingly, and ideally as an adjunct to appropriate allogeneic models.

## Author Contributions

All authors listed have made a substantial, direct, and intellectual contribution to the work and approved it for publication.

## Funding

This work was supported by a research grant from the National Heart, Lung, and Blood Institute of the NIH (R01HL148164), United States.

## Author Disclaimer

The content is solely the responsibility of the authors and does not necessarily represent the official views of the NIH.

## Conflict of Interest

GRH has consulted for Generon Corporation, NapaJen Pharma, Neoleukin Therapeutics. iTeos Therapeutics and has received research funding from Roche, Compass Therapeutics, Syndax Pharmaceuticals, Applied Molecular Transport and iTeos Therapeutics.

## Publisher’s Note

All claims expressed in this article are solely those of the authors and do not necessarily represent those of their affiliated organizations, or those of the publisher, the editors and the reviewers. Any product that may be evaluated in this article, or claim that may be made by its manufacturer, is not guaranteed or endorsed by the publisher.
